# Heart Diseases in Pregnancy: A Comprehensive Analysis of Maternal and Neonatal Outcomes

**DOI:** 10.7759/cureus.81678

**Published:** 2025-04-03

**Authors:** Ruchi Bansal, Nitesh Goyal, Chandrashekhar Shrivastava

**Affiliations:** 1 Obstetrics and Gynaecology, All India Institute of Medical Sciences, Raipur, IND; 2 Pulmonary, Critical Care, and Sleep Medicine, All India Institute of Medical Sciences, Raipur, IND

**Keywords:** ejection fraction and pregnancy, heart disease in pregnancy, maternal and neonatal outcomes, maternal cardiovascular health, peripartum cardiomyopathy, rheumatic heart disease (rhd)

## Abstract

Objective

This study aims to evaluate the prevalence, maternal and neonatal outcomes, and clinical implications of heart diseases in pregnancy.

Methods

A prospective observational study was conducted on pregnant women diagnosed with heart disease. Maternal and neonatal outcomes, including mortality, preterm birth, and mode of delivery, were assessed. Statistical comparisons were made based on ejection fraction (EF < 30% vs. EF ≥ 30%).

Results

Peripartum cardiomyopathy (n = 10, 27.78%) and rheumatic heart disease (n = 10, 27.78%) were the most prevalent cardiac conditions, followed by prosthetic valve disease and acyanotic congenital heart disease, each contributing to 11.11% (n = 4) of cases. A significant proportion of patients (n = 20, 55.56%) presented between 32 and 37 weeks of gestation. The study observed a maternal mortality rate of 13.89% (n = 5), with a stark contrast between women with an EF of <30% (n = 4, 33.33% mortality) and those with an EF of ≥30% (n = 1, 4.17% mortality). ICU admissions were required in 22.22% (n = 8) of cases, predominantly in women with an EF of <30% (n = 6, 50% vs. n = 2, 8.33%). Vaginal delivery was achieved in 66.67% (n = 24) of cases, while 27.78% (n = 10) underwent cesarean section. Among neonatal outcomes, 77.78% (n = 28) of infants were preterm, 52.78% (n = 19) had low birth weight (LBW), and 30.56% (n = 11) were small for gestational age (SGA). NICU admissions were recorded in 19.44% (n = 7) of cases, with a neonatal mortality rate of 5.56% (n = 2).

Conclusion

Heart diseases in pregnancy significantly impact both maternal and neonatal outcomes. Early detection and multidisciplinary management are crucial for improving prognosis and reducing complications.

## Introduction

Pregnancy serves as a physiological stressor for the body, leading to changes that may reveal underlying medical conditions. Often, these conditions are overlooked because their symptoms can be nonspecific or similar to normal pregnancy-related changes. The physiological changes during pregnancy can make diagnosing medical disorders more challenging, as they mimic or obscure the clinical signs of these conditions [1].

Medical disorders can interfere with the body’s ability to adapt to pregnancy, potentially leading to poor pregnancy outcomes and increased maternal and fetal mortality [2]. The rapid physiological changes that occur during pregnancy, childbirth, and the postpartum period can lead to periods of decompensation when the body fails to adjust. These phases of decompensation can put the mother and baby at risk, making careful monitoring and management essential during pregnancy.

Cardiac disease complicates 0.2-4% of all pregnancies in Western countries [3]. In developing countries like India, cardiac disease complicates 2% of pregnancies and contributes to about one-fifth of maternal deaths [4]. Morbidity and mortality due to congenital heart diseases have attained a plateau in developed countries [5]. However, acquired heart diseases are responsible for the rising trend of maternal deaths nowadays [6]. Heart failure, aortic dissections, myocardial infarction (MI), and arrhythmia are some of the presentations of acquired maternal heart disease during and after pregnancy [7]. In 2015, the United Kingdom maternal mortality report stated that more than 50% of maternal deaths occurred due to substandard health care, and half of such deaths could have been prevented. According to another research, it has been established that more than 25% of maternal deaths can be prevented if treating doctors consider cardiovascular disease in their differential diagnosis [5]. 

The presence of maternal heart disease increases complications during pregnancy and delivery. Ouyang et al., in their study on 112 pregnant patients with congenital heart disease, reported a 32.6% rate of adverse maternal outcomes, which included preterm delivery and postpartum hemorrhage (PPH) [8]. Heart diseases affect not only the pregnant mother but also the fetus since they increase the risk of spontaneous miscarriage; moreover, the fetus is also at increased risk of acquiring congenital heart disease [5]. While the risk of acquiring congenital heart disease in the general population is 1%, the risk is increased by 3-5% in fetuses born to mothers with congenital heart disease [9].

So, early detection and timely intervention of medical disorders that are not pregnancy-induced with the involvement of obstetricians, neonatologists, and nursing care are essential in reducing both maternal and neonatal complications. With this background, our study aimed to (1) identify the incidence of heart diseases that are not pregnancy-induced, 2) study the various maternal and fetal outcomes in pregnant patients with heart diseases, and (3) further categorize these maternal and fetal outcomes on the basis of ejection fraction (EF). This will result in better management of pregnant patients with heart diseases so that the best possible treatment and care can be provided, which will ultimately result in improved maternal and fetal outcomes.

## Materials and methods

This is a prospective type of study involving 36 pregnant patients with heart disease between March 2020 and April 2021. This study was conducted at the Postgraduate Institute of Medical Sciences (PGIMS), Rohtak, India, in the department of obstetrics and gynecology after approval from the institutional ethical committee.

Sampling

All consecutive pregnant patients with heart disease who were admitted were recruited in the study. Written informed consent was obtained from all the participants. Pregnant women with other preexisting medical conditions like diabetes, hypertension, anemia, and hypothyroid were excluded. Their complete history was noted, and relevant investigations were carried out. Different maternal, fetal, and neonatal outcomes were noted.

Definitions

Prematurity: gestational age < 37 weeks [10].

Low birth weight (LBW): birth weight < 2.5 kilograms [11].

Small for gestational age (SGA) neonates: birth weight < 10th percentile for gestational age [12].

Prolonged hospitalization: more than five days of hospital stay.

Statistical analysis

The data obtained was entered into a Microsoft Excel spreadsheet and analyzed using frequency and percentage. IBM SPSS Statistics for Windows, version 21.0 (IBM Corp., Armonk, NY) was used for further analysis of results.

In this study, qualitative variables were expressed as frequencies/percentages. The chi-square test and Fischer’s exact test were used to check the association between categorical variables. A p-value of <0.05 was considered statistically significant.

## Results

A total of 36 patients presented with heart disease over a period of 13 months. Table 1 shows the different types of heart disease in the studied subjects. Peripartum cardiomyopathy and rheumatic heart disease were most common contributing to 27.78% (n = 10) each in the studied subjects, followed by women with prosthetic valves, acyanotic congenital heart disease, dilated cardiomyopathy (DCMP), bicuspid aortic valves, hypertrophic obstructive cardiomyopathy (HOCM), MI, tetralogy of fallot (TOF), and Takayasu arteritis in descending order.

**Table 1:** Distribution of heart diseases into subcategories (n = 36)

Heart disease	n (%)
Peripartum cardiomyopathy	10 (27.78%)
Rheumatic heart disease	10 (27.78%)
Prosthetic valves	4 (11.11%)
Acyanotic congenital heart disease	4 (11.11%)
Dilated cardiomyopathy	2 (5.55%)
Bicuspid aortic valves	2 (5.55%)
Hypertrophic obstructive cardiomyopathy	1 (2.78%)
Myocardial infarction	1 (2.78%)
Tetralogy of fallot	1 (2.78%)
Takayasu arteritis	1 (2.78%)

Table 2 mentions the social and demographic details of the studied subjects. Seventeen patients (47.22%) were symptomatic at the time of presentation, and the rest were asymptomatic. The majority of patients (n = 20, 55.56%) presented were between 32 and 37 weeks of pregnancy. Most of the pregnant women with heart disease belonged to the urban population (n = 19, 52.78%). The mean age of the studied population was 26.55 ± 4.99 years, and 30 patients (83.33%) with heart disease were younger than 30 years. It was found that heart diseases were common in primigravida (n = 21, 58.33%). As per the modified Kuppuswamy scale, most of the women were categorized in the upper lower socioeconomic class (n = 20, 55.56%).

**Table 2:** Sociodemographic characteristics of the study population (n = 36)

Variables	n (%)
Nature of disease at the time of presentation	
Symptomatic	17 (47.22%)
Asymptomatic	19 (52.78%)
Period of gestation at the time of presentation	
<20 weeks	2 (5.55%)
20-24 weeks	1 (2.78%)
24-28 weeks	2 (5.55%)
28-32 weeks	3 (8.33%)
32-37 weeks	20 (55.56%)
>37 weeks	8 (22.22%)
Residence	
Rural	17 (47.22%)
Urban	19 (52.78%)
Age	
<25 years	17 (47.22%)
25-30 years	13 (36.11%)
30-35 years	5 (13.89%)
≥35 years	1 (2.78%)
Gravida	
Primigravida	21 (58.33%)
Multigravida	15 (41.67%)
Socioeconomic status	
Upper	0 (0%)
Upper middle	3 (8.33%)
Lower middle	11 (30.56%)
Upper lower	20 (55.56%)
Lower	2 (5.56%)

Table 3 classifies the different fetal and maternal outcomes in the studied population. Among various maternal outcomes, none of the patients had abruption and sepsis. PPH was seen in one (2.78%), prolonged hospitalization in 24 (66.67%), intensive care unit (ICU) admission in eight (22.22%), and maternal mortality in five (13.89%) patients. Most of the patients went into spontaneous labor (n = 27, 75%). Out of all the studied subjects, 24 (66.67%) patients delivered vaginally. Among fetal outcomes, prematurity was seen in 28 (77.78%) patients, LBW in 19 (52.78%), and SGA in 11 (30.56%) babies. Seven (19.44%) neonates were admitted to the neonatal ICU (NICU), out of which two (5.56%) neonates died. Stillbirth was noted in three (8.33%) babies.

**Table 3:** Maternal, fetal, and neonatal outcomes of the studied patients (n = 36) PPH, postpartum hemorrhage; ICU, intensive care unit; SGA, small for gestational age; NICU, neonatal ICU

Outcomes	n (%)
Maternal outcomes	
Abruption	0 (0%)
Sepsis	0 (0%)
PPH	1 (2.78%)
Prolonged hospitalization	24 (66.67%)
ICU admission	8 (22.22%)
Mortality	5 (13.89%)
Induced labor	7 (19.44%)
Spontaneous labor	27 (75%)
Vaginal delivery	24 (66.67%)
Cesarean delivery	10 (27.78%)
Fetal and neonatal outcomes	
Prematurity	28 (77.78%)
Low birth weight	19 (52.78%)
SGA	11 (30.56%)
NICU admission	7 (19.44%)
Mortality	2 (5.56%)
Stillbirth	3 (8.33%)

Table 4 shows a comparison of various maternal outcomes in the studied subjects with heart diseases upon division into two groups, namely group A and group B, on the basis of EF. Group A had an EF of <30%, and group B had an EF of ≥30%. Most of the patients came under group B (n = 24, 66.67%). More patients in group A (n = 6, 50%) were admitted to ICU admissions than group B, and the difference was statistically significant (p = 0.009). Maternal mortality was noted to be more for group A study group when compared with group B with a statistically significant difference (p = 0.098). Statistically significant differences could not be noted for the other variables, including labor induction, cesarean section, abruption, PPH, maternal sepsis, and prolonged hospitalization.

**Table 4:** Comparison of maternal outcomes among the two groups EF, ejection fraction; PPH, postpartum hemorrhage; ICU, intensive care unit

Criteria	Group A; EF < 30% (n = 12)	Group B; EF ≥ 30% (n = 24)	p-value
	n (%)	n (%)	
Induced labor	2 (16.67)	5 (20.83)	1
Caesarean delivery	5 (41.67)	5 (20.83)	0.188
Abruption	0 (0)	0 (0)	Not applicable
PPH	0 (0)	1 (4.17)	1
ICU admission	6 (50)	2 (8.33)	0.009
Maternal sepsis	0 (0)	0 (0)	Not applicable
Maternal mortality	4 (33.33)	1 (4.17)	0.098
Prolonged hospital stays	11 (91.67)	23 (95.83)	0.606

Prematurity, LBW, SGA, and neonatal mortality were common in babies born to mothers with heart disease and an EF of <30% (group A) (n = 11, 91.67%; n = 8, 66.67%; n = 5, 41.67%; and n = 1, 8.33%, respectively) as compared to other groups with an EF of ≥30% (group B) (n = 17, 70.83%; n = 11, 45.83%; n = 6, 25%; and n = 2, 8.33%, respectively), but no statistically significant difference could be established (Table 5). NICU admissions (n = 5, 20.83%) were more prevalent in neonates born to mothers having heart disease with an EF of ≥30% (group B). The incidence of stillbirths was the same in both groups.

**Table 5:** Comparison of fetal and neonatal outcomes among the two groups EF, ejection fraction; SGA, small for gestational age; NICU, neonatal ICU

Criteria	Group A; EF < 30% (n = 12)	Group B; EF ≥ 30% (n = 24)	p-value
	n (%)	n (%)	
Prematurity	11 (91.67)	17 (70.83)	0.224
Low birth weight	8 (66.67)	11 (45.83)	0.302
SGA	5 (41.67)	6 (25)	0.306
Still birth	1 (8.33)	2 (8.33)	1
NICU admission	2 (16.67)	5 (20.83)	1
Mortality	1 (8.33)	1 (4.17)	1

## Discussion

Cardiac disease in pregnant patients presents significant challenges in both maternal and fetal care. Pregnancy itself induces substantial physiologic changes in the cardiovascular system, including increased blood volume, elevated cardiac output, and altered vascular resistance. These changes are necessary to support the growing fetus, but they can place additional strain on the heart and can exacerbate pre-existing cardiac conditions. As the pregnancy progresses, the body’s increasing demands can result in complex interactions between the heart, circulation, and the developing fetus, which require careful management to mitigate risks for both maternal and fetal well-being. Understanding the balance between these physiological changes and underlying cardiac conditions is crucial for optimizing outcomes for both mother and child [13].

Understanding how heart disease manifests and progresses during pregnancy is crucial for managing these patients effectively. Regular monitoring, close observation, and tailored treatment strategies are essential. Managing pre-existing cardiac conditions requires multidisciplinary care involving obstetricians, cardiologists, and sometimes neonatologists to ensure both maternal and fetal health. Furthermore, counselling and education are key, as awareness of potential complications and the importance of regular follow-up care are crucial to cardiovascular health [14].

In our study, it was found that the most common heart disease encountered was rheumatic heart disease and peripartum cardiomyopathy, which affected 27.78% (n = 10) each in the studied subjects, followed by pregnancy with prosthetic valves and acyanotic heart diseases with equal affection of 11.11% (n = 4) each. Although the findings of our study are significant, they cannot be generalized to the whole population due to the smaller sample size. These diseases were followed by DCMP, bicuspid aortic valves, HOCM, TOF, and MI in descending order of frequency. Kambhampati et al., in their study on pregnant women with heart disease, also found that rheumatic heart disease was most common (55%), followed by cyanotic and acyanotic congenital heart diseases (32%), and other conditions constituted 13% [15].

At the time of presentation, we found that the mean age was 26.55 ± 4.99 years, and 83.33% (n = 30) of women with heart disease were less than 30 years of age. Similar results were reported in a study by Subbaiah et al., where they found that the mean age of presentation of patients with heart disease in pregnancy was 26.9 ± 4.09 [16]. Similarly, Salam et al., in their study, also found that a maximum number of patients (74.5%) were between 20 and 30 years of age [17]. In our study, it was found that heart diseases were more prevalent in primigravida (n = 21, 58.33%). Similar results were found by Salam et al., where the majority of the patients were primigravidae or primipara (60%) [17].

Our study also compared various maternal, fetal, and neonatal outcomes in heart diseases, which were divided into two groups on the basis of EF (<30% and ≥30%). The outcomes were much worse for group A (EF < 30%) than group B (EF ≥ 30%). But the difference is statistically significant only for ICU admissions (50% vs. 8.33%) and maternal mortality (33.33% vs. 4.17%) with a p-value of 0.009 and 0.098, respectively. More patients in group A landed up in cesarean section as compared to group B (41.67% vs. 20.83%) as either they were directly taken for cesarean section in view of their heart disease or those left for vaginal delivery had more of incidences of fetal distress leading to cesarean section, which can be explained by presence of heart disease with EF less than 30% in them. Similar results were obtained in a study carried out by Salam et al., where they found that the majority of the women delivered by cesarean section (n = 33, 36.7%) [17]. They also reported four maternal deaths [17]. Similarly, in a systematic review by van Hagen and Roos-Hesselink, they found that perinatal mortality rates were higher in pregnancies complicated by maternal heart disease, particularly in cases of cyanotic CHD and left heart obstruction [18]. The findings of our study are also consistent with Siu et al., who reported that maternal complications occurred in 13% of pregnancies, with left heart obstruction and poor functional class being one of the major risk factors [19]. In a meta-analysis by Regitz-Zagrosek et al., they found that heart failure was the leading cause of maternal mortality in women with heart disease, further emphasizing the importance of close monitoring for signs of decompensation during pregnancy [20]. Similarly, a study by Drenthen et al. demonstrated that women with an EF of <30% had significantly higher rates of adverse maternal events [21]. Hence, the results of our study highlight the importance of individualized delivery planning in pregnant women with cardiovascular disease, particularly with low EF.

For the neonatal outcomes, there was no significant statistical difference in outcome of any of the variable, but the data was poorer for most of the variables in group A (EF < 30%) with a prematurity of 91.67% (n = 11), LBW of 66.67% (n = 8), SGA of 41.67% (n = 5), stillbirth of 8.33% (n = 1), NICU admission of 16.67% (n = 2), and expired neonatal of 8.33% (n = 1) compared to group B with percentages of 70.83% (n = 17), 45.83% (n = 11), 25% (n = 6), 8.33% (n = 2), 20.83% (n = 5), and 4.17% (n = 1), respectively. This proves that EF has an essential role in defining the fetomaternal outcome in heart disease during pregnancy. Similarly, in a study by Siu et al., they found that the neonatal complications were associated with poor functional class or cyanosis and left heart obstruction [19]. Premature birth and SGA birth weight were the most common events reported in their study. Fetal and neonatal death complicated eight and seven pregnancies, respectively [19]. Therefore, the current study’s findings reinforce the critical role of cardiac function, particularly left ventricular EF, in determining maternal as well as neonatal and fetal outcomes.

The present study emphasizes the need for comprehensive maternal cardiac assessment and multidisciplinary care, including cardiologists, obstetricians, and neonatologists, to optimize pregnancy outcomes. Early identification of cardiac dysfunction, particularly reduced EF, can aid in risk stratification and timely intervention.

Even though the present study has provided valuable insights, there are certain limitations. First, the sample size is relatively small; hence, the findings of our study shall be verified on a large number of patients before generalizing its findings to the broader population. Second, the study was conducted in a single tertiary care hospital in North India, and regional variations in healthcare access and management may influence outcomes. Lastly, potential confounding factors such as socioeconomic status, pre-existing comorbidities, gestational age at presentation, and adherence to treatment protocols were not extensively analyzed, which could impact the interpretation of results. Future studies addressing these limitations may enhance our understanding of heart disease in pregnancy and improve patient management.

## Conclusions

This study provides valuable insights into the burden of heart disease in pregnancy and its associated maternal and neonatal outcomes. The findings reinforce existing evidence that cardiac disease significantly impacts pregnancy outcomes, with higher morbidity and mortality rates in women with severe cardiac dysfunction. A multidisciplinary approach, early screening, and individualized management strategies are essential to improving maternal and fetal health in pregnant women with heart disease.
